# Implications of Isolated Para-Aortic Lymph Node Metastasis in Endometrial Cancer: A Large-Scale, Multicenter, and Retrospective Study

**DOI:** 10.3389/fmed.2021.754890

**Published:** 2021-10-21

**Authors:** Wenting Li, Jie Jiang, Yu Fu, Yuanming Shen, Chuyao Zhang, Shuzhong Yao, Congjian Xu, Min Xia, Ge Lou, Jihong Liu, Bei Lin, Jianliu Wang, Weidong Zhao, Jieqing Zhang, Wenjun Cheng, Hongyan Guo, Ruixia Guo, Fengxia Xue, Xipeng Wang, Lili Han, Xia Zhao, Xiaomao Li, Ping Zhang, Jianguo Zhao, Jiezhi Ma, Qin Yao, Xiaohang Yang, Yingyu Dou, Zizhuo Wang, Jingbo Liu, Yong Fang, Kezhen Li, Beibei Wang, Gang Chen, Xiaodong Cheng, Chaoyang Sun, Beihua Kong

**Affiliations:** ^1^Cancer Biology Research Center (Key Laboratory of the Ministry of Education), Tongji Medical College, Tongji Hospital, Huazhong University of Science and Technology, Wuhan, China; ^2^Department of Gynecology and Obstetrics, Tongji Medical College, Tongji Hospital, Huazhong University of Science and Technology, Wuhan, China; ^3^Department of Obstetrics and Gynecology, Cheeloo College of Medicine, Qilu Hospital, Shandong University, Jinan, China; ^4^School of Medicine, Women's Hospital, Zhejiang University, Hangzhou, China; ^5^Department of Gynecologic Oncology, Sun Yat-sen University Cancer Center, Guangzhou, China; ^6^Department of Obstetrics and Gynecology, The First Affiliated Hospital of Sun Yat-sen University, Guangzhou, China; ^7^Department of Gynecology, Obstetrics and Gynecology Hospital of Fudan University, Shanghai, China; ^8^Department of Gynecology and Obstetrics, The Affiliated Yantai Yuhuangding Hospital of Qingdao University, Shandong, China; ^9^Department of Gynecology Oncology, Harbin Medical University Cancer Hospital, Harbin, China; ^10^Department of Obstetrics and Gynecology, Shengjing Hospital Affiliated to China Medical University, Shenyang, China; ^11^Peking University People's Hospital, Beijing, China; ^12^Division of Life Sciences and Medicine, The First Affiliated Hospital of University of Science and Technology of China, University of Science and Technology of China, Hefei, China; ^13^Department of Gynecologic Oncology, Guangxi Medical University Cancer Hospital, Guangxi, China; ^14^The First Affiliated Hospital of Nanjing Medical University, Nanjing, China; ^15^The Third Hospital of Peking University, Beijing, China; ^16^Department of Gynecology and Obstetrics, The First Affiliated Hospital of Zhengzhou University, Zhengzhou, China; ^17^Department of Gynecology and Obstetrics, Tianjin Medical University General Hospital, Tianjin, China; ^18^Department of Gynecology and Obstetrics, Xin Hua Hospital, Shanghai JiaoTong University School of Medicine, Shanghai, China; ^19^Department of Gynecology, People's Hospital of Xinjiang Uygur Autonomous Region, Urumqi, China; ^20^Department of Gynecology and Obstetrics, Development and Related Disease of Women and Children Key Laboratory of Sichuan Province, Key Laboratory of Birth Defects and Related Diseases of Women and Children, Ministry of Education, West China Second Hospital, Sichuan University, Chengdu, China; ^21^Department of Gynecology and Obstetrics, The Third Affiliated Hospital, Sun Yat-sen University, Guangzhou, China; ^22^Department of Gynecology, The Second Hospital of Shandong University, Jinan Shandong, China; ^23^Department of Gynecologic Oncology, Tianjin Central Hospital of Gynecology and Obstetrics, Affiliated Hospital of Nankai University, Tianjin, China; ^24^Tianjin Clinical Research Center for Gynecology and Obstetrics, Branch of National Clinical Research Center for Gynecology and Obstetrics, Tianjin, China; ^25^Department of Obstetrics and Gynecology, Xiangya Third Hospital, Central South University, Changsha, China; ^26^Department of Obstetrics and Gynecology, The Affiliated Hospital of Qingdao University, Qingdao, China

**Keywords:** isolated para-aortic, lymph node metastasis, endometrial carcinoma, clinical significance, retrospective study

## Abstract

**Objective:** To systematically evaluate lymph node metastasis (LNM) patterns in patients with endometrial cancer (EC) who underwent complete surgical staging, which included systematic pelvic and para-aortic lymphadenectomy.

**Methods:** Four thousand and one patients who underwent complete surgical staging including systematic pelvic and para-aortic lymphadenectomy for EC were enrolled from 30 centers in China from 2001 to 2019. We systematically displayed the clinical and prognostic characteristics of patients with various LNM patterns, especially the PLN-PAN+ [para-aortic lymph node (PAN) metastasis without pelvic lymph node (PLN) metastasis]. The efficacy of PAN+ (para-aortic lymph node metastasis) prediction with clinical and pathological features was evaluated.

**Results:** Overall, 431 of the 4,001 patients (10.8%) showed definite LNM according to pathological diagnosis. The PAN+ showed the highest frequency (6.6%) among all metastatic sites. One hundred fourteen cases (26.5%) were PLN-PAN+ (PAN metastasis without PLN metastasis), 167 cases (38.7%) showed PLN+PAN-(PLN metastasis without PAN metastasis), and 150 cases (34.8%) showed metastasis to both regions (PLN+PAN+). There was also 1.9% (51/2,660) of low-risk patients who had PLN-PAN+. There are no statistical differences in relapse-free survival (RFS) and disease-specific survival (DSS) among PLN+PAN-, PLN-PAN+, and PLN+PAN+. The sensitivity of gross PLNs, gross PANs, and lymphovascular space involvement (LVSI) to predict PAN+ was 53.8 [95% confidence interval (CI): 47.6–59.9], 74.2 95% CI: 65.6–81.4), and 45.8% (95% CI: 38.7–53.2), respectively.

**Conclusion:** Over one-fourth of EC patients with LMN metastases were PLN-PAN+. PLN-PAN+ shares approximate survival outcomes (RFS and DSS) with other LNM patterns. No effective clinical methods were achieved for predicting PAN+. Thus, PLN-PAN+ is a non-negligible LNM pattern that cannot be underestimated in EC, even in low-risk patients.

## Introduction

Endometrial cancer is one of the most common gynecologic cancers with a rising incidence (1). It was estimated that 417,000 women were diagnosed, and 97,300 women died of this disease in 2020 (2). According to the International Federation of Gynecology and Obstetrics (FIGO), the lymph node metastasis (LNM) status of patients with endometrial cancer (EC) is an essential reference for assigning a pathological stage, guiding adjuvant treatment, and determining prognostic value (3). The 5-year overall survival (OS) rate of patients with either pelvic lymph node metastasis (PLN+) or para-aortic lymph node metastasis (PAN+) is roughly 57–66% (4).

The FIGO 2009 Staging stratified Stage IIIC into Stage IIIC1 (PLN+) and Stage IIIC2 (PAN+ ± PLN+) by the presence of para-aortic lymph node metastasis, indicating a worse prognosis with PAN involvement (5–7). The most common LNM patterns are well-recognized as PLN+PAN- and PLN+PAN+, since the reported incidence of isolated para-aortic lymph node metastasis (PLN-PAN+) is merely around 1–3% in EC (8, 9). With the further investigation on LNM patterns in EC, the incidence of PLN-PAN+ among patients with LNM differs widely in recent studies, varying from 6 to 46.2% (5, 10–23). The adequacy and extent of lymphadenectomy in EC remain controversial worldwide, dominantly on the extent of para-aortic lymph node dissection (24, 25). The increasing incidence of PLN-PAN+ would partially strengthen the insight for para-aortic lymph node dissection. Also, the prognostic impact of PLN-PAN+ among patients with LNM remains unclear. Thus, a large sample size of EC cases is urgent to obtain the objective incidence and prognostic implications of PLN-PAN+.

It is well-acknowledged that myometrial invasion, degree of differentiation, histological type, tumor size, and tumor site are used as references for guiding surgical planning (26–29). Also, the National Comprehensive Cancer Network (NCCN) guidelines make recommendations regarding sentinel lymph nodes (SLNs) in EC. It is worth exploring further whether these clinical features and detective techniques could play a role in evaluating PAN+(30), especially PLN-PAN+.

In this study, we aim to describe the incidence and characteristics of PLN-PAN+ in a large-scale sample of patients with EC from multicenter across China and seek to evaluate the prognostic impact of PLN-PAN+ on recurrence and disease-specific survival. Moreover, the accuracy of the current methods used to identify techniques for predicting PAN+ would be tested.

## Materials and Methods

### Study Design and Participants

To gain unique data on EC in China, we formed the Chinese Endometrial Carcinoma Consortium (CECC), which includes 30 academic centers from different regions of China, and generated a database from January 1, 2000, to December 31, 2019.

In this study, we included over-18-year-old patients with primary EC who had undergone comprehensive surgical staging: hysterectomy, bilateral adnexectomy, pelvic washing, and pelvic and para-aortic lymphadenectomy (31). Systematic pelvic lymphadenectomy included the resection of common iliac, external iliac, internal iliac, obturator, sacral, medial deep inguinal, and lateral deep inguinal nodes; para-aortic lymphadenectomy included the systematic resection of all nodes from the precaval, laterocaval, interaortocaval, preaortic, and lateroaortic areas up to the renal veins. Patients were excluded if they had treatment for their endometrial cancer (such as radiotherapy, chemotherapy, or hormonal therapy), had previously undergone retroperitoneal surgery or lymphadenectomy, or had other concurrent primary cancer. This study was approved by Institutional Review Boards in all CECC centers.

### Data Sources and Measurement

Patient data were collected from computerized patient records in each center. Clinical features, included age at diagnosis, histology, grading, type of pathology, pathology-related information [status of lymph node metastasis, specific location, myometrial invasion, lymphovascular space involvement (LVSI), cervical involvement, para-uterine involvement, and other extra-uterine metastasis], time to recurrence, cause of death, and OS (in months), were collected.

Relapse-free survival is defined as the time from surgery to relapse. Disease-specific survival (DSS) is defined as the time from surgery to death due to EC. Patients known to be alive or lost to follow-up at the time of analysis were reviewed at the last follow-up visit (September 14, 2019).

### Statistical Methods

Descriptive statistics were used for patient demographics and disease characteristics. Demographic and clinical characteristics were compared by LNM status. Student's unpaired *t*-tests (age) were performed to compare two samples of continuous variables, and chi-square or Fisher's exact test was performed to compare the proportion of the two samples. RFS and DSS were calculated according to Kaplan-Meier curves, and differences among subgroups were evaluated by stratified log-rank tests. Hazard ratio (HR), 95% CI, and *p*-values for DSS/RFS among each group were estimated using univariate Cox proportional hazards models. The accuracy of predicting PAN+ was evaluated by sensitivity and the Youden index. Statistical significance was set at 0.05. Statistical analyses were performed with IBM SPSS 26.0 (SPSS Inc., Chicago, IL, United States).

## Results

### Patient Clinical Characteristics

**Figure 1:**
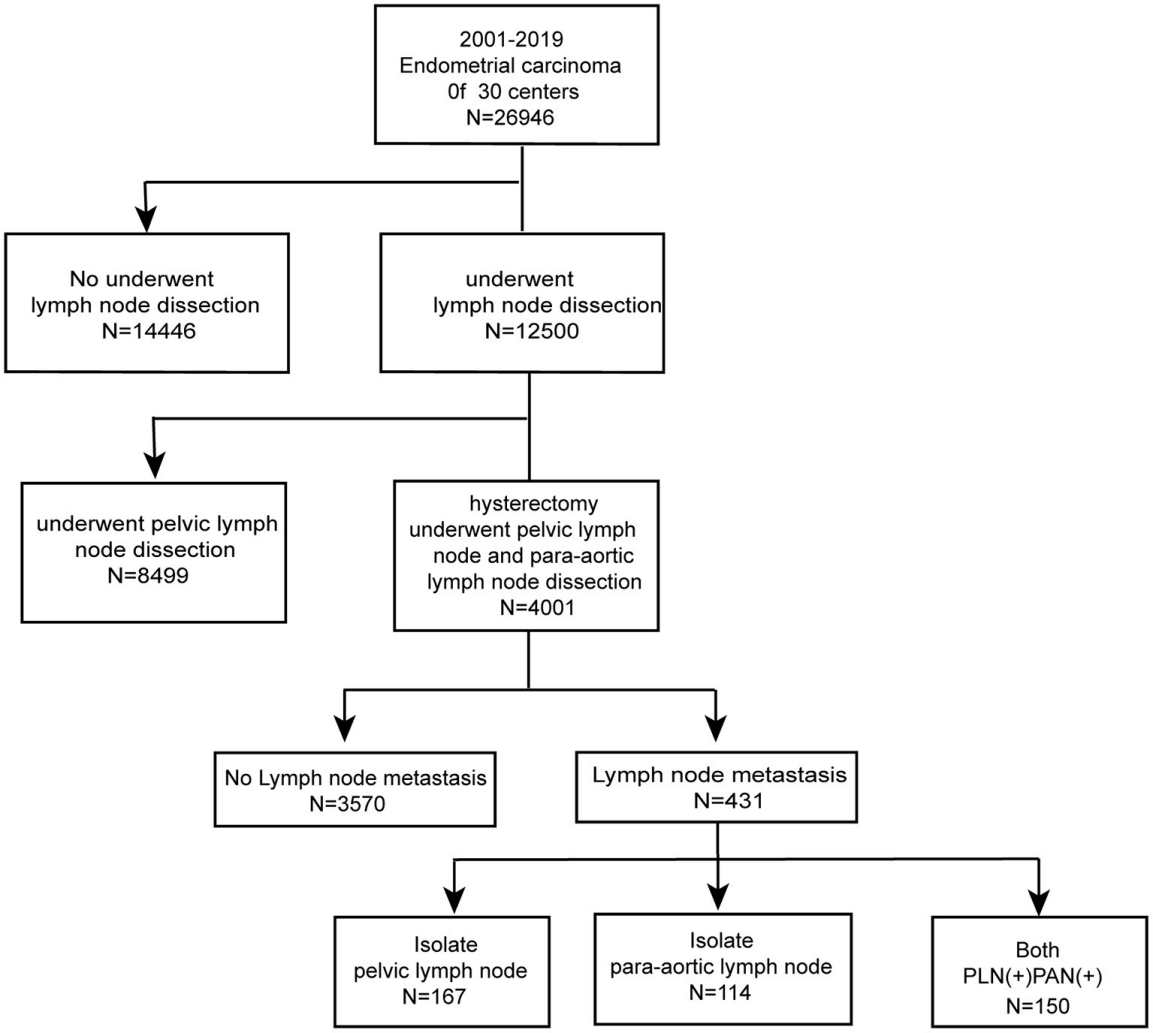
Study cohort diagram.

Figure 1 shows the number of patients assessed at every stage in the study. Among 26,946 patients, 4,001 (14.8%) underwent hysterectomy and simultaneous PAN dissection and PLN dissection. The median age was 55 years (range, 22–83 years). Referring to the status of LNM, 3,570 (89.2%) cases were recognized as negative LNM, whereas 431 (10.8%) cases were considered positive LNM. In order to investigate the characteristics of LNM status, we collected clinical and pathological records for comparison (Table 1). Samples with missing values were excluded from statistical analysis. As expected, the portions of histological grades were significantly different in the two groups (*P* < 0.001), whereas fewer patients in positive LNM cases were well-differentiated (61 vs. 80.3%, *P* < 0.001). Also, the incidence of cervical involvement showed a slight increase in the positive LNM group (12.6 vs. 8.9%, *P* = 0.046). No significant differences were observed in the two groups on age, histology, myometrial invasion, LVSI, para-uterine involvement, and other extra-uterine metastasis.

**Table 1:** Characteristics of 4,001 patients with endometrial cancer (EC) who underwent PLND and PAND.

**Characteristic**	**No Lymph node metastasis** ***N*** **= 3,570 (%)**	**Lymph node metastasis** ***N*** **= 431 (%)**	***p*** **-value**
**Ages**			0.361
Median, year (range)	54 (22–79)	55 (22–81)	
Not reported	7		
**Grade**			<0.001
1	1,073 (31.9)	73 (17.1)	
2	1,625 (48.4)	187 (43.9)	
3	607 (18.1)	155 (36.4)	
Undifferentiated	54 (1.6)	11 (2.6)	
Not reported	211	5	
**Histology**			0.916
Endometrioid	3,129 (87.6)	377 (87.5)	
Non-endometrioid	441 (12.4)	54 (12.5)	
**Myometrial invasion**			0.532
Endometrium only	232 (7.0)	25 (6.5)	
Inner half	2,192 (66.8)	269 (69.7)	
Outer half	855 (26.8)	92 (23.8)	
Not reported	291	45	
**Lymphovascular space involvement**			0.729
No	2,763 (90.1)	327 (90.3)	
Yes	277 (9.1)	35 (9.7)	
Not reported	530	69	
**Cervical involvement**			0.046
NO involvement	2,846 (91.1)	325 (87.4)	
Mucous layer	74 (2.3)	10 (2.7)	
Cervical stroma	205 (6.6)	37 (9.9)	
Not reported	445	59	
**Para-uterine involvement**			
No	3,190 (97.1)	383 (97.2)	0.911
Yes	95 (2.9)	11 (2.8)	
Not reported	285	37	
**Other extra-uterine metastasis**			0.681
No	3,435 (96.4)	416 (96.7)	
Yes	130 (3.6)	14 (3.3)	
Not reported	5	1	

*PLND, pelvic lymph node dissection; PAND, para-aortic lymph node dissection*.

*P-values are based on the Chi-square test or Students t-test between two groups in the clinical characteristics*.

### Incidence and Distribution of Different Metastatic Patterns in EC

**Figure 2:**
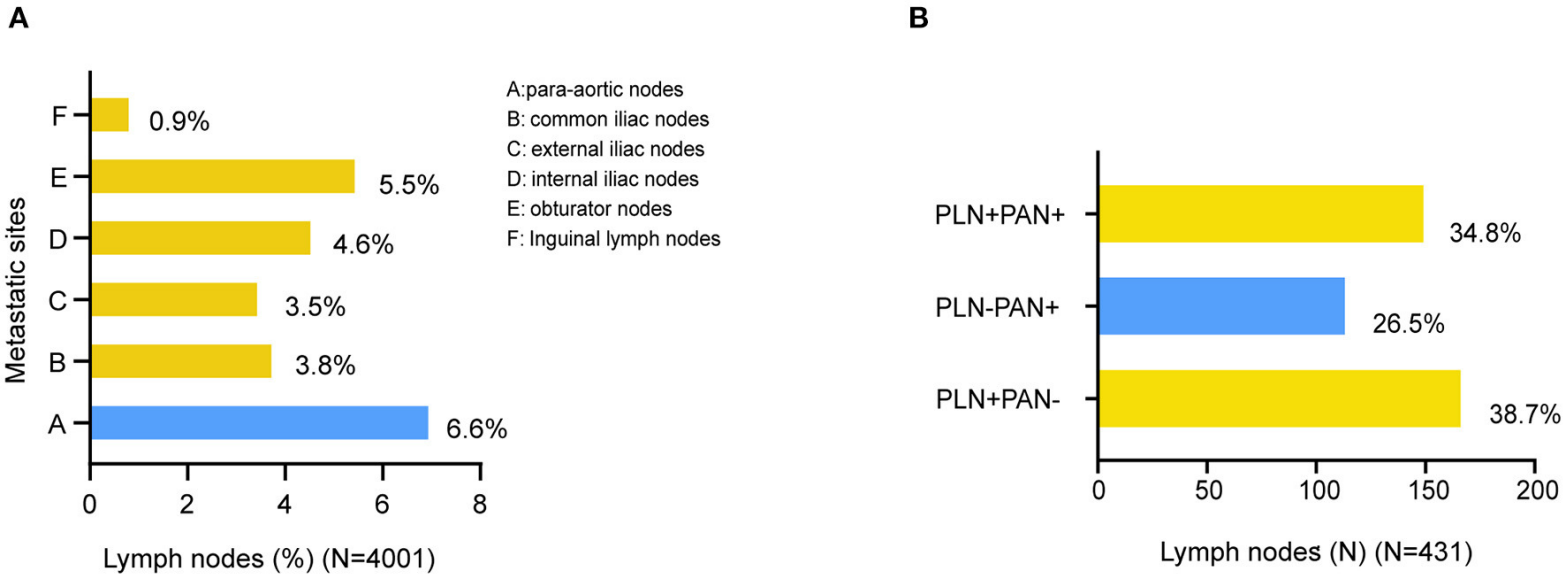
Distribution of LNMs in EC. **(A)** Distribution of metastatic site in surgically staged. **(B)** Distribution of metastatic patterns.

According to FIGO, we categorized LNM into three metastatic patterns: PLN+PAN-, PLN-PAN+, and PLN+PAN+. The overall LNM rate was 10.8% (431/4,001) among the patients who underwent both PLN and PAN dissection. The incidence of PLN+PAN-, PLN-PAN+, and PLN+PAN+ was 4.2 (167/4,001), 2.8 (114/4,001), and 3.8% (150/4,001), respectively, in our cohort. Of 431 patients who were positive for LNM, 38.7% (167/431) cases were PLN+PAN-, 26.5% (114/431) cases were PLN-PAN+, and 34.8% (150/431) cases showed PLN+PAN+ (Figure 2B).

The distribution of metastatic sites was summarized among all the 4,001 patients (Figure 2A). The most prevalent site of LNM was PAN+ (6.6%, 264/4,001), followed by obturator nodes (5.5%, 222/4,001), internal iliac nodes (4.6%, 184/4,001), common iliac nodes (3.8%, 153/4,001), and external iliac nodes (3.5%, 142/4,001). The rarest location of LNM was inguinal lymph nodes (0.9%, 35/4,001).

### Prognostic Impacts of Different Metastatic Patterns

**Figure 3:**
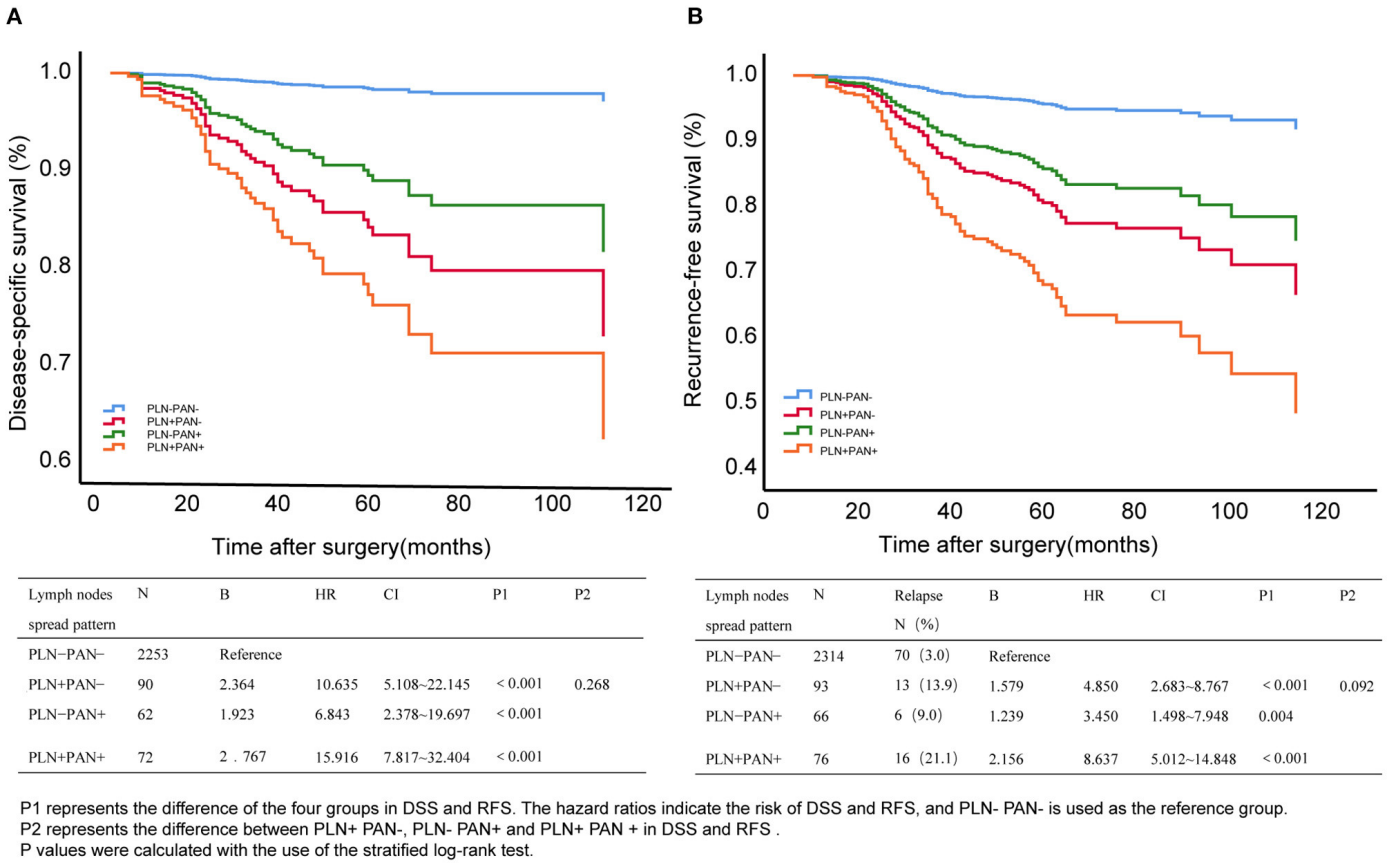
Disease-specific survival (DSS) **(A)** and relapse-free survival (RFS) **(B)** of different metastatic patterns in EC. P1 represents the difference between the four groups in DSS and RFS. The hazard ratios indicate the risk of DSS and RFS, and PLN-PAN- is used as the reference group. P2 represents the difference between pelvic lymph node metastasis without para-aortic lymph node metastasis (PLN+PAN-), PAN metastasis without PLN metastasis (PLN-PAN+), and both PLN and PAN metastasized (PLN+PAN+) in DSS and RFS. *p*-values were calculated by the stratified log-rank test.

Here, we enrolled double prognostic events, relapse, and death to comprehensively evaluate clinical outcomes in patients with different LNM patterns. The Kaplan–Meier curves display the DSS and RFS in Figure 3. Referring to the LNM negative group, all LNM patterns were associated with poor outcomes, among which PLN+PAN+ showed the highest risk for RFS (HR = 8.637, 95% CI = 5.012–14.848, *P* < 0) and DSS (HR = 15.916, 95% CI = 7.817–32.404, *P* < 0.001). Among the different LNM patterns, PLN+PAN+ indicated the poorest trends for DSS and RFS, followed by PLN+PAN-, and then PLN-PAN+. Remarkably, the clinical outcomes of PLN-PAN+ were comparable with those of the other two common patterns, evaluated by log-rank test (DSS: *P* = 0.268, RFS: *P* = 0.092).

### Accuracy of Different Methods for Predicting Metastasis in PANs

Currently, the most commonly used intraoperative prediction methods for PANs are combined with high-risk factors (gross LNs, LVSI) and SLN by lead surgeons. SLN was not evaluated in this study because of a lack of adequate data. Current treatment guidelines recommend that patients with endometrioid adenocarcinoma, superficial myometrial infiltration (<50%), and well-differentiation be the low-risk population and should be omitted for systematic lymph node dissection. However, our study found that 7.4% (198/2,660) of low-risk patients had LMN and that 1.9% (51/2660) had PLN-PAN+, as shown in Table 2. Furthermore, we evaluated the effectiveness of gross PLNs, gross PALNs, and LVSI on the prediction of PAN+. The sensitivity of gloss PALNs (gloss PALNs: 74.2%) is slightly higher than that of the others (gloss PLNs: 53.8%, LVSI: 45.8%), while specificity is the lowest (gloss PALNs: 61.8%). The Youden indexes of gloss PLNs, gloss PALNs, and LVSI were 0.392, 0.360, and 0.39, respectively. The results indicated that gloss PLNs, gloss PALNs, and LVSI could not predict PAN+ well-before PAN desertion (Table 3).

**Table 2:** Characteristics of different lymph node metastasis (LNM) patterns in EC with different risk factors.

**RISK**	**No. Cases**	**PLN-PAN- No. Cases (%)**	**PLN+PAN+ No. Cases (%)**	**PLN+PAN- No. Cases (%)**	**PLN-PAN+ No. Cases (%)**
Grade 1–2 endometrioid, myoinvasion ≤50%	2,660	2,462 (92.6)	52 (1.9)	95 (3.6)	51 (1.9)
Grade 1–2 endometrioid, myoinvasion>50%	36	28 (77.8)	4 (11.1)	1 (2.8)	3 (8.3)
Grade 3 endometrioid	605	492 (81.3)	49 (8.1)	35 (5.8)	29 (4.8)
Non-endometrioid	482	375 (77.8)	44 (9.1)	34 (7.1)	29 (6.0)

**Table 3:** Diagnostic indices for predicting para-aortic lymph node metastasis by gross nodes and lymphovascular space involvement (LVSI).

	**Para-aortic lymph node metastases**		**Total**	**Sensitivity % (95% CI)**	**Specificity % (95% CI)**	**Youden index**
	**Positive**	**Negative**				
**Gross pelvic nodes**						
Positive	142	547	689	53.8 (47.6–59.9)	85.4 (84.2–86.4)	0.392
Negative	122	3,188	3,310			
Total	264	3,735	3,999			
**Gross Para-aortic nodes**						
Positive	95	187	282	74.2 (65.6–81.4)	61.8 (57.4–66.1)	0.360
Negative	33	303	336			
Total	128	490	618			
**LVSI**						
Positive	88	224	312	45.8 (38.7–53.2)	93.2 (92.1–93.9)	0.39
Negative	104	2,987	3,091			
Total	192	3,211	3,403			

## Discussion

With an extensive collection of medical records from 30 centers, we comprehensively analyzed LNM patterns in 4,001 individuals who had undergone simultaneous PLN and PAN dissection. In this study, we demonstrated the non-negligible incidence and terrible prognostic impact of PLN-PAN+ among patients with EC.

The overall LNM rate was 10.8% among the patients who underwent both PLN and PAN dissection, which was similar to the rate of 9.9–21.6% derived from the previous study (5, 10–23). In our cohort, the incidence of PLN-PAN+ was 2.8%, which was in close agreement with 3% in the research of Kumar et al. (9) and 2.8% in Todo et al. (5). Moreover, regarding the distribution of LNM sites, our findings show that metastasis occurs most frequently in PAN+, 6.6%. The prevalence distribution is consistent with another study by Odagiri et al. (15). Together, these results indicate that PLN-PAN+, a key site in a postoperative stage in EC, is critical for solving postoperative personalized adjuvant treatment.

Unexpectedly, we uncovered that over one-fourth (26.5%, 114/431) of individuals with positive LNM showed PLN-PAN+ in this large-sample study. For comparison, we further calculated the incidence of PLN-PAN+ from other research studies, which were all based on a small sample size, and found that it varied from 6 to 46.2%, (5, 10–23). Over the past decade, the widely used Mayo criteria (18) have provided well-recognized risk factors for LNM in EC. According to their protocol, lymphadenectomy is not recommended for the low-risk group. However, in our study, 1.9% of patients in the low-risk group identified by Mayo criteria, which is not small probability events, were PLN-PAN+. Therefore, PLN-PAN+ with low-risk patients is rare but may occur. These results make us rethink the high-risk factors for LNM in EC.

Delaying recurrence and improving the prognosis of ECs are the primary concerns of patients and gynecologic oncologists. In this study, we discussed the value of PLN-PAN+ in recurrence and prognosis. Referring to non-LNM, LNM demonstrated sharp decreases in both RFS and DSS. Although PLN+PAN+ showed a worse prognostic trend, no statistically significant differences were laid on clinical outcomes among the different patterns of metastasis: PLN-PAN+, PLN+PAN-, and PLN+PAN+. These results were consistent with the study of Guo et al. (23), indicating that relapse and prognosis are strongly associated with all the LNM patterns. During our postoperative follow-up, we found that para-aortic lymph node recurrence occurred in seven patients. Notably, six of the seven patients did not undergo PAN dissection, and the other one patient did not undergo systematic lymph node dissection, indicating that PAN is a high-risk location for tumor metastasis. Thus, PLN-PAN+ is also an important issue that deserves further attention from gynecologic oncologists. Moreover, the “jumping metastasis” in the lymphatic drainage pathway makes LNM status more difficult to predict in EC.

The pre- and intraoperative identification of patients at high risk for PLN-PAN+ is challenging (18). The current intraoperative assessment of LNM status mainly uses high-risk factors, gross LNs, or cervical tracer-labeled SLNs (32). Our findings demonstrated the limitations of using LVSI and intraoperative gross LNs in the assessment of PAN+. According to the Sentinel Lymph Node Biopsy vs. Lymphadenectomy for Intermediate- and High-Grade Endometrial Cancer Staging (33) prospective cohort study, SLN biopsy had an acceptable diagnostic accuracy of positive LN in high-grade EC with a high risk of LNM. However, the potential risk of missing PLN-PAN+ remains one of the main concerns and criticisms (30, 34), because SLN labeling on cervical tracer injection may not adequately map the PAN area (35). Then, additional fundal site injections of SLN labeling are needed to improve the assessment of PANs (36, 37). Multinu et al. found that ultrastaging and pathologic review can identify occult pelvic lymph node metastases and reduce the prevalence of true PLN-PAN+ (38). Clinicopathological and intraoperative observations might not be enough to predict PLN-PAN+ in the pre- and intraoperative stages. New biomarkers may be urgently needed for identifying PLN-PAN+ pre-operatively, which will be fulfilled along with the developing understanding of EC on the molecular level.

Our study has both limitations and strengths. First, this retrospective study led to a selection bias. Second, the molecular classification of EC could play a role in prognosis and lymph node metastasis patterns, but due to national conditions in China, the lack of such data prevents us from making a more adequate assessment. The strength mainly lies in the large population from multiple superior hospitals with standardized diagnosis and therapy procedures. To the best of our knowledge, this study represents the largest series reporting PLN-PAN+ cases.

## Conclusion

In this nationwide, a large number and retrospective study, the PLN-PAN+ pattern was comparable to the other two metastasis patterns (PLN+PAN- and PLN+PAN+) in terms of incidence rate and prognostic impact. Clinical methods and molecular biomarkers are urgently needed to identify patients at high risk for PLN-PAN+ in the following studies.

## Data Availability Statement

The raw data supporting the conclusions of this article will be made available by the authors, without undue reservation.

## Ethics Statement

Written informed consent was obtained from the individual(s) for the publication of any potentially identifiable images or data included in this article.

## Author Contributions

GC, CS, and BW worked on the design of the study. Data collection was conducted by YS, CZ, SY, CX, MX, GL, JihL, BL, JW, WZ, JieZ, WC, HG, RG, FX, XW, LH, XZ, XL, PZ, JiaZ, JM, QY, YFa, and KL. XY, YD, ZW, and JinL were responsible for the cohort follow-up. WL, YFu, and JJ performed data extraction and analysis and wrote the manuscript. BK and XC supervised the whole study. All authors contributed to the article and approved the submitted version.

## Conflict of Interest

The authors declare that the research was conducted in the absence of any commercial or financial relationships that could be construed as a potential conflict of interest.

## Publisher's Note

All claims expressed in this article are solely those of the authors and do not necessarily represent those of their affiliated organizations, or those of the publisher, the editors and the reviewers. Any product that may be evaluated in this article, or claim that may be made by its manufacturer, is not guaranteed or endorsed by the publisher.

## Abbreviations

CECC: Chinese Endometrial Carcinoma Consortium
DSS: disease-specific survival
EC: endometrial cancer
FIGO: International Federation of Gynecology and Obstetrics
HR: Hazard ratio
LNM: lymph node metastasis
LVSI: lymphovascular space involvement
NCCN: National Comprehensive Cancer Network
OS: overall survival
PAN: para-aortic lymph node
PAN+: para-aortic lymph node metastasis
PLN: pelvic lymph node
PLN+: pelvic lymph node metastasis
PLN-PAN+: PAN metastasis without PLN metastasis
PLN+PAN-: PLN metastasis without PAN metastasis
PLN+PAN+: both PLN and PAN metastasized
RFS: relapse-free survival
SLN: sentinel lymph node.
